# Immunogenicity of trimeric autotransporter adhesins and their potential as vaccine targets

**DOI:** 10.1007/s00430-019-00649-y

**Published:** 2019-12-01

**Authors:** Arno Thibau, Alexander A. Dichter, Diana J. Vaca, Dirk Linke, Adrian Goldman, Volkhard A. J. Kempf

**Affiliations:** 1 Institute for Medical Microbiology and Infection Control, University Hospital, Goethe-University, Paul-Ehrlich-Str. 40, 60596 Frankfurt am Main, Germany; 2 Section for Genetics and Evolutionary Biology, Department of Biosciences, University of Oslo, Oslo, Norway; 3 Astbury Centre for Structural Molecular Biology, School of Biomedical Sciences, University of Leeds, Leeds, UK; 4 Molecular and Integrative Biosciences Program, University of Helsinki, Helsinki, Finland

**Keywords:** Trimeric autotransporter adhesins, Immunogenicity, Vaccination, Pathogenicity, Virulence

## Abstract

The current problem of increasing antibiotic resistance and the resurgence of numerous infections indicate the need for novel vaccination strategies more than ever. In vaccine development, the search for and the selection of adequate vaccine antigens is the first important step. In recent years, bacterial outer membrane proteins have become of major interest, as they are the main proteins interacting with the extracellular environment. Trimeric autotransporter adhesins (TAAs) are important virulence factors in many Gram-negative bacteria, are localised on the bacterial surface, and mediate the first adherence to host cells in the course of infection. One example is the *Neisseria* adhesin A (NadA), which is currently used as a subunit in a licensed vaccine against *Neisseria meningitidis*. Other TAAs that seem promising vaccine candidates are the *Acinetobacter* trimeric autotransporter (Ata), the *Haemophilus influenzae* adhesin (Hia), and TAAs of the genus *Bartonella*. Here, we review the suitability of various TAAs as vaccine candidates.

## Introduction

**Fig. 1:**
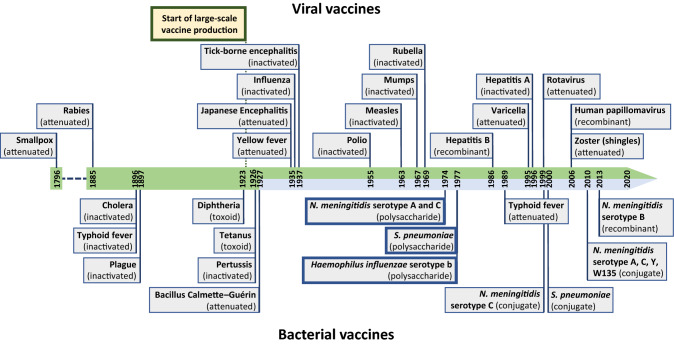
Timeline of the development of human vaccines showing the scarcity of newly developed bacterial vaccines since 1927. Viral vaccines are shown above, while bacterial vaccines are shown below the timeline. Only the first developed vaccine against each viral or bacterial species is depicted (except for typhoid fever, *N. meningitidis* spp. and *S. pneumoniae* because of the different vaccine compositions). Not all invented, produced or updated vaccine formulation are included, only the major developments. Noteworthy is that vaccines against only three bacterial agents (*N. meningitidis* spp., *S. pneumoniae,* and *H. influenzae*) were developed since 1927 (light blue part in timeline) [1, 135, 251–253]

Vaccination against human pathogens was first introduced in medicine in 1796 by Edward Jenner (Fig. 1). He realised that milkmaids who had suffered earlier from cowpox were not infected by smallpox, demonstrating that the inoculated vaccinia virus leads to immunological protection against the variola virus [1]. Nowadays, vaccination represents a life-saving, scientifically accepted, and low-cost procedure to efficiently avoid human infections [2, 3]. Very recently, the national German government announced a program to increase the rate of measles vaccination in the population [4]. Although prophylaxis of infections by vaccination is very effective, there is, unfortunately, only a limited number of licensed vaccines available, most of which target viruses (Fig. 1). Current vaccines do, therefore, not cover most of the infectious diseases and, on top of that, many diseases for which vaccination strategies would be desirable, are on a resurgence (e.g., whooping cough) [5–10]. Novel vaccine formulations or alternative approaches must be investigated and a promising way forward is the use of recombinant vaccine components, developed from, e.g., reverse vaccinology approaches [3, 11]. However, the development of vaccines against emerging infectious diseases including Gram-negative bacteria decelerated in the last decades. Noteworthy is that new vaccines against only three bacterial agents were developed since 1927 (Fig. 1). In this review, we focus on the immunogenicity and vaccine candidacy of trimeric autotransporter adhesins (TAA) as one particular group of outer membrane proteins (OMPs) of Gram-negative bacteria [12–17]

Principally, the most important conditions necessary to be an effective vaccine component are (i) the in vivo expression of surface epitopes, (ii) a high strain coverage, and (iii) immunogenicity and induction of a protective immune response in the host [18, 19]. In general, bacterial membrane proteins such as TAAs perform numerous important functions in pathogenesis, of which the first interaction with the extracellular environment in the mammalian host is of crucial importance. The extent of virulence of pathogenic organisms depends on various characteristics of both the organism itself (i.e., capacity of entering, infiltrating, and spreading through the host) and the host defence (i.e., immune status and metabolic conditions) [20–22]. It has become evident that TAAs play a prominent role in bacterial pathogenicity, where quick adaptation to changing conditions is crucial. As such, the modular composition of TAAs and their highly repetitive nature makes it possible for rapid adaptation to the host to occur [16, 23]. Moreover, attachment of bacteria via TAAs to the host is the first and absolutely required step in the infection process. Therefore, TAAs are highly suitable as vaccine candidates [12, 23–25].

## Trimeric autotransporter adhesins

**Fig. 2:**
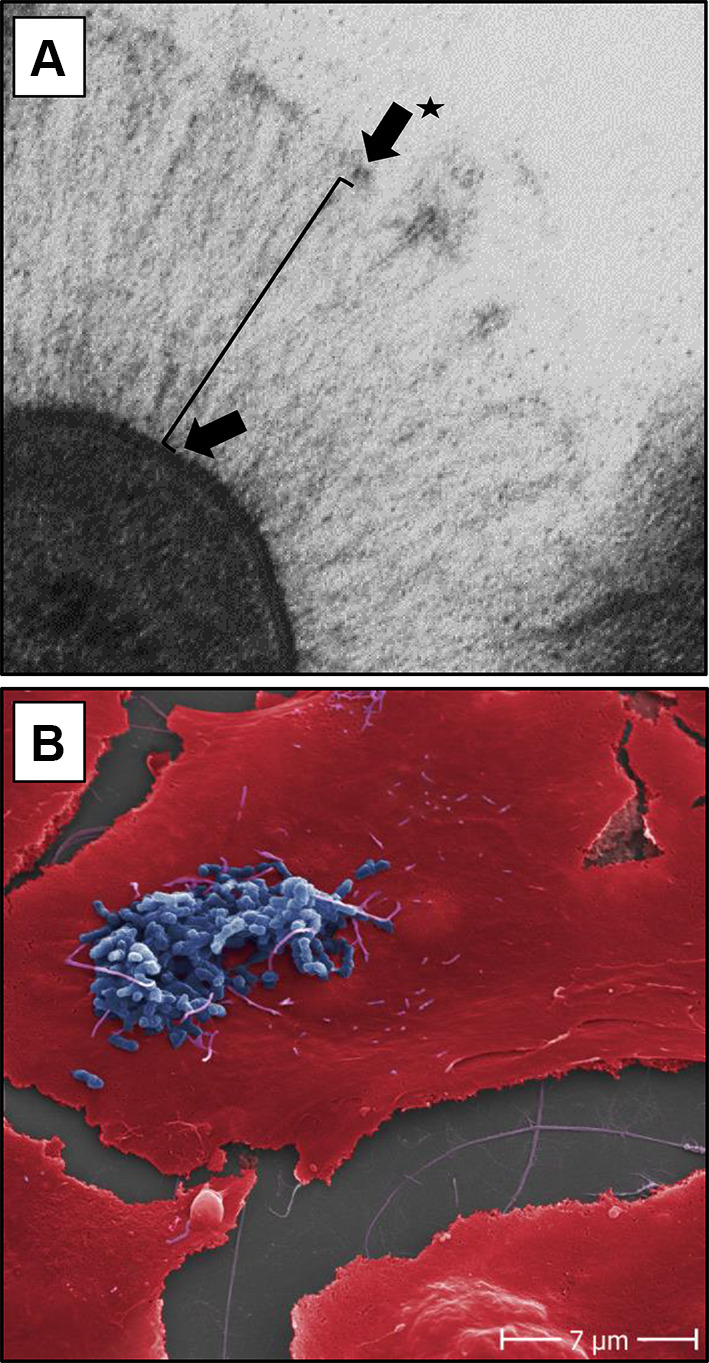
Electron microscopy of *B. henselae* adhesin A and adherence of *B. henselae* Marseille to human endothelial cells. **a** ‘Lollipop-like’ surface structure of the long filamentous BadA with the globular N-terminal head domain (arrow with star), followed by the passenger domain consisting of a neck/stalk domain (black line) and the membrane anchor (not visible) spanning the outer membrane (arrow). **b***B. henselae* Marseille (blue coloured) adhering to the surface of human umbilical vein endothelial cells (red coloured) 30 min upon infection. Scale bare: 7 µm

TAAs are a family of obligate homotrimeric, non-fimbrial, non-pilus bacterial adhesins that have numerous biological functions such as bacterial autoagglutination, binding to extracellular matrix (ECM) proteins and host cells, and the induction of distinct host cell responses. They are widespread in α-, β-, and γ-proteobacteria and primarily ensure the initial adhesion to specific molecular components of both abiotic and biotic surfaces (Fig. 2b) [16, 23, 26]. Former and alternative designations for TAAs are non-fimbrial adhesins (NFAs) and oligomeric coiled-coil adhesins (Ocas) [27–29] of which the latter refers to the presence of coiled coils in the structure of prototypical members of this class [30].

In general, all TAAs share a common lollipop-like surface structure (Fig. 2a). The C-terminal anchor domain (translocation unit) forms a 12 stranded ß-barrel transmembrane domain followed by a passenger domain consisting of a neck/stalk domain and an N-terminal head domain. The head domain often has a globular structure and is responsible for the majority of the TAA’s biological functions [24, 29, 31]. The anchor domain, which defines the family, is conserved in all TAAs and ensures the autotransporter activity [16, 24, 30].

Type V secretion systems are autotransporters containing a ß-barrel transmembrane domain [32]. Five different type V secretion systems have so far been identified (type Va, Vb, Vc, Vd, and Ve), all of which are used to transport proteins across the outer membrane in Gram-negative bacteria [26, 33, 34]. The type Vc secretion system is also termed TAA. Several models for the autotransporter mechanism exist, but the details remain unknown [32, 34, 35]. After translocation, the passenger domain remains covalently attached to the anchor domain (Fig. 2a). Previously, it was thought that the translocation of the passenger domain across the outer membrane occurred without any external source of free energy (ion gradients, chaperone proteins, or adenosine triphosphate) [27]. However, recent experimental research on TAAs has demonstrated that the ß-barrel assembly (Bam) complex is likely to catalyse the translocation of the passenger domain across the outer membrane [36], on top of its known function to integrate the ß-barrel anchor domain into the outer membrane. This theory challenges the current ‘autotransporter’ hypothesis, however, does not change the fact that translocation is driven by the free energy of protein folding. The Bam complex consists of five proteins and catalyses the insertion of almost every ß-barrel in the outer membrane of Gram-negative bacteria [33, 34, 37–40].

## The use of type V(c) secretion in vaccinology

Even though the exact secretion mechanism of TAAs is still unclear, the Vc secretion system is a potentially valuable feature in the development of multivalent recombinant bacterial vector vaccines [41–44]. For instance, it was suggested for HIV-1 envelope glycoprotein subunits (e.g., gp120) that soluble stabilised trimers generate a stronger immunogenic response in mice compared to monomeric exterior immunogenic glycoproteins [45, 46]. This may be due to the higher stability of trimers in vivo, the presence of multiple, cross-linked epitopes and, in this case, the more faithful representation of the functional envelope glycoprotein complex [45]. In contrast to the type Va secretion system, the type Vc secretion system manages to expose stable trimeric polymers on the outer membrane of Gram-negative bacteria, showing its potential in future vaccine development [23].

In case of the type Va secretion system, autotransport of recombinant heterologous expressed proteins has already been demonstrated to optimise antigen delivery in oral live-attenuated vaccine strains, increasing the immunogenicity and improving the specific immune response [47–49]. Furthermore, Jong et al. emphasized the potential of autotransporter adhesins as a valuable platform to display antigens for the development of multivalent recombinant vector vaccines by successfully expressing various heterologous antigens via the *Escherichia coli* autotransporter Hbp (type Va secretion system) both in *E. coli* and in an attenuated *Salmonella enterica* serovar Typhimurium vaccine strain [50].

## Reverse vaccinology and outer membrane vesicles

A more recent vaccine delivery platform is the use of outer membrane vesicles (OMV) because of their high immunogenicity and virulence during infection [42, 51–53]. Recombinant vaccine antigens, such as TAAs, that can be added on OMVs, are primarily selected via reverse vaccinology, which includes in silico genome screening for open reading frames that likely encode for antigenic OMPs [53–55]. OMVs do not replicate, which makes them safer and thus more attractive candidates as vaccine components [56, 57]. However, they do not guarantee broad strain coverage and often mediate protection only against closely related strains [53, 58, 59]. In addition, lipopolysaccharides (LPS) are abundantly present in OMVs causing numerous inflammatory side effects in OMV-based vaccines [60].

## TAAs as vaccine (sub)units

The most extensively investigated TAA is the *Yersinia* adhesin A (YadA) from *Yersinia enterocolitica*, the prototypical example of this class of adhesins [16, 26, 30]. Furthermore, *Neisseria* adhesin A (NadA) from *Neisseria meningitidis* is already one of the main vaccine antigens in the respective multicomponent vaccine, 4CMenB [61]. Other interesting TAAs and potential vaccine antigens are, *inter alia, Haemophilus influenzae* adhesin (Hia) (*H. influenzae*) [62], *Acinetobacter* trimeric autotransporter (Ata) (*A. baumannii*) [63], *Salmonella* adhesin A (SadA) (*S. enterica*) [64], and the ubiquitous surface proteins (UspA1 and UspA2) of *Moraxella catarrhalis* [65]. The proven immunogenicity of several TAAs makes them a potential target for vaccine development and their use in clinical diagnosis [23, 66]. Below, we discuss the vaccinology prospects of most of the well-studied TAAs (Table 1).

**Table 1:** Immunogenicity of trimeric autotransporter adhesins and their potential as vaccine (sub)units

Genus	Species	Licensed vaccine available?	TAA	Est. MW (kDa)^a^	Strain prevalence	UniProt accession no.	Immunogenicity	Protective properties	Considered as vaccine antigen	References
*Yersinia* spp.	*Y. enterocolitica*	No	YadA	47	High prevalence in both strains with few genomic and phenotypic variants	P31489	Proven: serum poly- and monoclonal antibodies in rabbit and mice, but no mucosal antibodies in mice	Partly proven in mice	No (not by itself)	[38, 71, 84, 92, 95]
	*Y. pseudo*-*tuberculosis*	No								
*Neisseria* spp.	*N. meningitidis*	Yes	NadA	43	High prevalence (50–75%) in disease-associated isolates; six NadA variants and 89 distinct *nadA* allele sequences exist	Q8KH85	Proven: strong antibody response in mice and bactericidal serum and mucosal antibodies in humans	Proven: in an infant rat infection model and in humans	Yes [licensed vaccine containing NadA available (4CMenB)]	[25, 98, 110, 114, 116, 118, 124]
			NhhA	62	Highly conserved in all meningococcal strains; some isolated MenB strains only (partially) express monomeric NhhA	Q7DDJ2	Proven: serum antibodies in humans and serum bactericidal antibodies in mice (in conjugation with other antigens)	NA	Yes	[52, 97, 123, 125]
*Haemophilus* spp.	*H. influenzae*	Yes: targeting Hib No: NTHi and remaining encapsulated *H. influenzae*	Hia	114	Only present in 25% of NTHi clinical isolates	Q48152	Proven: opsonophagocytic serum antibodies in guinea pigs and mice	Not proven (but strongly suggested)	Yes (in combination with HMW1, HMW2 and NTHi OMVs)	[62, 133, 136, 139, 145]
			Hsf	243	Present in all encapsulated serotypes and a subset of NTHi	P71401	Proven: serum polyclonal antibodies in rabbit	NA	No	[129, 132]
	*H. ducreyi*	No	DsrA	30	High prevalence in both *H. ducreyi* clonal populations with varieties in the DsrA passenger domain	Q9K2H6	proven: serum antibodies in swine and in mice	Proven: in swine and mice	Yes	[66, 147, 149, 150]
*Acinetobacter* spp.	*A. baumannii*	No	Ata	250	High prevalence (78%) in monophyletic *A. nosocomialis*, *A. seifertii* and *A. baumannii*	A3M3H0 K7ZP88	Proven: serum, bactericidal and opsonophagocytic antibodies in mice and rabbit antiserum	Proven: reduction in lung bacterial burdens in mice	Yes	[55, 63, 155, 158, 159]
*Moraxella* spp.	*M. catarrhalis*	No	UspA1	83,5	High prevalence of *uspA1* (97%) and *uspA2* (83%); strain-specific gene differences and variable phenotypes	A0A3Q9GAK7 Q9XD52	Proven: serum and mucosal antibodies in children and adults; bactericidal antibodies detected in mouse and guinea pig anti-sera	Proven: pulmonary clearance of bacteria in immunised mice	Yes (in the past)	[18, 163, 168, 172, 173, 175–177, 179]
			UspA2	59,5		B5L5X1 Q9XD55				
*Escherichia* spp.	*E. coli* (EHEC, STEC, EAEC, ExPEC and VTEC)	No	EibA	42	NA	Q9LA60	NA	NA	No	[186–189, 197–199]
			EibC	53	NA	Q9LA56				
			EibD	54	NA	Q9MCI8				
			EibE	52	NA	Q9LA53				
			EibF	49	NA	Q8VW24				
			EibG	54	Low prevalence in STEC (15%)	Q0EAF1				
	*E. coli* (STEC*)*		Saa	56	High prevalence in specific LEE-negative STEC strains^b^	Q93F81	Proven: polyclonal serum antibodies in mice	NA	Yes (for LEE-negative STEC strains)	[182]
	*E. coli* (UPEC and ExPEC)		UpaG	178	Low prevalence in UPEC (21%), mostly related to *E. coli* B2 and D phylogenetic groups and frequently associated with ExPEC	A0A0H2VCA1	Proven: serum antibodies in mice and rabbit	Proven: in mice after active and passive immunisation targeting ExPEC	Yes	[183, 184, 195]
	*E. coli* (EHEC)		EhaG	160	NA	Q7DJ60	Proven: serum antibodies in rabbit	No protective properties	No	[204]
*Salmonella* spp.	*S. enterica* (serovar Typhimurium)	Yes	SadA	147	High prevalence in *S. enterica* strains and highly conserved sequence	Q8ZL64	Proven: IgG response in mice	Modest protection in mice	No (not by itself)	[64, 204, 209, 210]
*Bartonella* spp.	*B. bacilliformis*	No	BbadA (BrpA)	130	NA	A0A3G2T987 A1UT92	NA	NA	Yes	[211, 225, 226]
			BbadB (BrpB)	132		A1URN1				
			BbadC (BrpC)	57		A1URM8				
	*B. henselae*	No	BadA	328	highly conserved in *B. henselae*; variable length in passenger domain	Q5MWV9	Proven: serum antibodies in rabbit and IgG antibodies in patient sera	NA	No	[24, 232, 238, 240, 241]
	*B. quintana*	No	Vomp A	101	Heterogeneity in *vomp* gene locus from *B. quintana* human isolates; highly conserved genes; variably expressed	Q64HS9	Proven: immunoreactive in human sera infected with *B. quintana*	NA	Yes	[212, 213, 220]
			Vomp B	109		Q64HS8				
			Vomp C	104		Q64HS7	NA		No	
			Vomp D	99		Q64HT0				

*NA* not assessed

^a^Monomeric

^b^Locus of enterocyte effacement (LEE) pathogenicity island

### *Yersinia* spp. TAA

YadA is a TAA present on the bacterial surface of *Y. enterocolitica* and *Yersinia pseudotuberculosis*. *Yersinia pestis* harbours the *yadA* gene, but the TAA is not expressed due to a frameshift mutation in the *yadA* gene [67].

Infections of *Y. enterocolitica* and *Y. pseudotuberculosis* are caused by the ingestion of contaminated food or water and can cause acute enteritis and lymphadenitis (pseudoappendicitis) in the gastrointestinal tract [68, 69], sometimes followed by sequelae such as arthritis and septicaemia [70]. Subsets of *Y. pseudotuberculosis* are the causative agent of, e.g., Far East scarlet-like fever [69].

Currently, there are no licensed vaccines targeting *Y. pestis* and *Y. pseudotuberculosis* [71]. Earlier human vaccines comprising live-attenuated *Yersinia* strains or killed whole-cell bacteria [72] often caused severe side reactions or proved to be too reactogenic, respectively [72–75]. Some vaccines are in clinical trials (e.g., rF1-V and RYpVax) and seem the ideal approach to overcome more outbreaks of *Y. pestis* by providing pre-exposure prophylaxis to combat infection for individuals with a high risk of exposure [71]. Important to note is, however, the fact that *Y. pestis* does not express YadA precluding its use as a potential plague vaccine candidate.

Successful first attempts to develop effective vaccines against *Y. enterocolitica* were established using different *Yersinia* proteins. In 1996, Noll and Autenrieth used heat shock proteins (*Yersinia* HSP60) with IL-12 as adjuvant in their vaccine development [76]. They suggested that microbial heat shock proteins would be promising vaccine candidates. Palmer et al. demonstrated the ability of *Y. enterocolitica* to modulate the immune response via OMPs [77]. More recently, the effective use of a bivalent fusion protein consisting of immunologically active regions of *Y. pestis* LcrV (i.e., a 35 kDa secreted protein that mediates the transport effector proteins into the host cell [71, 75]) and YopE proteins gave mice immunogenic protection upon delivery of lethal *Y. enterocolitica* [78]. New screening approaches for the development of vaccine candidates are still necessary, for instance, in vivo signature-tagged mutagenesis to target genes for novel virulence factors [79] or the use of reverse vaccinology to screen for antigenic OMPs.

The immunodominant YadA of *Y. enterocolitica* has a monomeric molecular weight of approximately 47 kDa [31, 38] and the *yadA* gene is located on the 64-75 kb *Yersinia* virulence plasmid (pYV) [80, 81]. Although discovered in 1981 as ‘protein 1’ [82, 83], YadA is still investigated to unravel its complex structure, to clarify the autotransporter mechanism and to identify its biological functions [16, 36].

Between the different *Yersinia* strains, highly homologous YadA proteins exist [84]. Different pathogenic and virulence functions are attributed to YadA in *Y. enterocolitica* and *Y. pseudotuberculosis* [80, 85]. For example, a short amino acid sequence was identified within the N-terminal head domain of YadA from *Y. pseudotuberculosis* that mediates uptake in human cells and promotes binding to the ECM protein fibronectin [84]. Later, it was shown that a similar stretch also exists in distinct strains of *Y. enterocolitica*, but only in those of serotype O:9. There, the stretch was crucial for efficient binding of the serum protein vitronectin [86]. Furthermore, the YadA-passenger domain confers serum resistance and is important for the pathogenicity of *Y. enterocolitica* [30, 87]. In addition, Schütz et al. demonstrated that the trimeric stability of YadA is crucial for full pathogenicity of *Y. enterocolitica* [88]. YadA itself induces the production of proinflammatory cytokines, including interleukin-8 (IL-8) and this process is triggered via the adhesion to β1-integrins [89, 90].

Some research has been carried out towards the immunogenicity of YadA. For example, poly- and monoclonal antibodies against YadA were obtained and antigens were identified upon immunisation with live bacteria [91–93]. According to Tahir et al., it is of interest to use purified YadA or killed *Y. enterocolitica* instead of live bacteria in vaccines [94]. They indicated that live *Y. enterocolitica* can prevent the host from recognising other than N-terminal epitopes of YadA. Finally, in 2017, Tsugo et al. immunised mice subcutaneously either with recombinantly expressed YadA (group 1), with inactivated *Y. pseudotuberculosis* strongly expressing YadA (group 2), or just with phosphate-buffered saline (group 3—control). Survival rates after exposure to pathogenic *Y. pseudotuberculosis* were 100% (group 1), 60% (group 2), and 0% (group 3), respectively [95]. However, the recombinantly expressed YadA proteins did not induce mucosal immunity as measured by IgG secretion. The authors concluded that YadA shows promising results as a vaccine component, but more research towards its safety, immunogenicity, and protective properties is necessary [95].

### *Neisseria meningitidis* TAAs

The *Neisseria* adhesin A (NadA) and the *Neisseria* Hia/Hsf homologue A (NhhA) are both OMPs belonging to the class of TAAs. Both adhesins are present on certain genetic lineages of the Gram-negative bacterium *Neisseria meningitidis* [16, 96, 97].

*Neisseria meningitidis* is a human-specific Gram-negative pathogen and is the causative agent of meningococcal meningitis and sepsis [98, 99] with over 500,000 meningococcal cases each year worldwide [61, 100–102]. Twelve meningococcal serogroups have been classified based on their capsular polysaccharides. The serogroups A, B, C, W-135, X, and Y are most associated with invasive diseases [102–104]. Currently, serogroup B meningococci (MenB) causes most of the epidemic and endemic meningococcal diseases and is responsible for one-third of the meningococcal infections [52, 105]. Despite antibiotic treatment and partially effective vaccines, the progression of the disease is quick and has high mortality rates (5–15%) [98, 100].

In general, three types of meningococcal vaccines are available: polysaccharide vaccines, polysaccharide–protein conjugated vaccines and vaccines based on OMPs (developed via reverse vaccinology) [13, 103]. In the case of polysaccharide vaccines, bi-, tri-, or tetravalent vaccines exist, of which only the tetravalent vaccine is still available in Europe [103]. Effective tetravalent conjugate polysaccharide vaccines, combination vaccines, or monovalent vaccines against *N. meningitidis* serogroups A, C, W-135 and Y have been available since the early 1990s, are licensed, and are in clinical use [103]. The MenB capsular polysaccharide, however, shows high similarities with *N*-acetyl neuraminic acid on the surface of human fetal neural tissues and is, therefore, poorly immunogenic [25, 106, 107]. A protective capsular polysaccharide-based vaccine against serogroup B is thus not being pursued [52, 98]. Nevertheless, recently, two protein-based MenB vaccines were approved and licensed in several countries [105]. In 2013, the four component MenB vaccine 4CMenB (Bexsero^®^), using an OMV and three recombinant proteins [two protein–protein fusions and a single antigen (NadA)] was approved by the European Union (EU) [13, 103, 108]. Later, the recombinant protein vaccine MenB-FHbp was licensed in the USA (2014) and the EU (2018). It contains two variants of the meningococcal surface protein factor H-binding protein (FHbp) [58, 109].

#### *Neisseria meningitidis* adhesin A

NadA is a phase-variable ca. 43 kDa OMP of which the expression is mainly regulated by the transcriptional regulator NadR [96, 110, 111]. NadA plays a crucial role in the attachment of *N. meningitidis* to epithelial cells via ß1-integrins and in its subsequent invasion during the infection process [112, 113]. NadA is immunogenic, induces a protective bactericidal response, and has self-adjuvanting activity [114–116]. Furthermore, two genetically distinct groups of NadA exist that share only 46–50% identity and that do not show immunological cross reactivity [117, 118]. Group I (sharing ca. 95% sequence identity) consists of the variants NadA1, NadA2, and NadA3, while Group II (sharing ca. 90% sequence identity) consists of the variants NadA4, NadA5, and NadA6 [98, 118, 119]. Variants are classified based on their main variant group and small mutations [118, 119]. For example, NadA4 is mainly associated with carriage strains [98, 119, 120]. The crystal structures of NadA5 and NadA3 are available and provide valuable information for further investigations on their biological functions and on the effectiveness and structure of NadA as a vaccine antigen [61, 98, 121].

The *nadA* gene is present in approximately 30% of *N. meningitidis* isolated strains and in 75% of hypervirulent *N. meningitidis* serogroup B lineages [112, 117, 122]. Comanducci et al. demonstrated via dot-blot hybridization and PCR that 47% of 150 representatives of disease-associated isolates harbour the *nadA* gene [25]. In case of commensal strains derived from healthy carriers, *nadA* is present in 16.2% of 154 isolates [117].

Currently, NadA is the only TAA that is used as a component in a licensed vaccine, as NadA3 is a major antigen in the multicomponent vaccine 4CMenB [61, 96, 98]. In 2002, Comanducci et al. were the first to propose NadA as a vaccine candidate against MenB by demonstrating the strong inducement of antibodies upon immunisation of mice with NadA and showing protective features in an infant rat model [25]. Two years later, NadA was proven to be the only antigen out of 23 selected meningococcal proteins that elicits a strong antibody response in convalescent infant patients suffering from a meningococcal infection [123]. In 2006, Giuliani et al. described a universal vaccine against MenB that makes use of 5 antigens discovered by reverse vaccinology and aluminium hydroxide as an adjuvant [124]. In 2013, the 4CMenB vaccine was approved by the EU, promptly followed by a vaccination campaign in infants in the UK [13, 96]. Summarised, NadA and its discovery via reverse vaccinology, its analysis as an essential pathogenicity factor of *N. meningitidis*, and the further development as a vaccine component serve as a role model to expedite the development of TAA-based vaccines.

#### *Neisseria meningitidis* Hia/Hsf homologue A

NhhA was the first vaccine candidate against MenB and was described using whole genome sequencing to identify possible vaccination targets [54]. NhhA shows high similarities with the TAAs Hia and *Haemophilus* surface fibril (Hsf) of *Haemophilus influenzae*, is immunogenic in humans in conjugation with other antigens (e.g., TbpA, Omp85, or NspA), and facilitates bacterial attachment to host epithelial cells during infection by binding heparan sulphate and laminin [97, 99, 125, 126]. Furthermore, NhhA mediates serum resistance, induces macrophage apoptosis, reduces phagocytosis, and protects the bacteria against complement-mediated killing [99, 127]. Moreover, the *nhha* gene is highly conserved in all meningococcal strains [19, 97].

All these features suggest that NhhA is a promising vaccine candidate [23, 111]. Peak et al. immunised mice with OMVs containing various NhhA constructs, demonstrating protective properties of truncated NhhA against heterologous NhhA-expressing *N. meningitidis* strains [97]. A later study showed an enhanced immunogenicity against NhhA when its membrane anchor domain was coupled to the *Moraxella* IgD-binding protein providing a more effective vaccine [52].

However, it was found that a subset of clinical isolated MenB strains only (partially) express the monomeric form of NhhA, caused by a single natural mutation (glycine to aspartic acid) in the C-terminal passenger domain. Accordingly, loss in trimerization, surface exposure and adhesive features was observed. These findings question the vaccine candidacy of NhhA because of the need for broad strain coverage [128].

### *Haemophilus* spp. TAAs

Two different TAAs are expressed on the outer membrane of *H. influenzae*, *H. influenzae* adhesin (Hia), and *H.* surface fibril (Hsf) [129, 130].

*Haemophilus influenzae* is a human specific, Gram-negative pathogen categorised into two different groups, the polysaccharide encapsulated (serotypes a–f), and the unencapsulated group often referred to as non-typeable *H. influenzae* (NTHi) [129, 131, 132]. *H. influenzae* serotype b (Hib) encapsulated strains are considered most virulent and are a major agent of respiratory tract systemic infections. Infections can lead to acute epiglottitis, sepsis, acute meningitis, and pneumonia. NTHi mainly causes local diseases such as bronchitis, otitis media, and sinusitis [131, 133, 134].

Current vaccines are mainly against the most virulent Hib. The earlier polysaccharide-based vaccines showed only short-term protection for children under 18 months after various trials were undertaken in 1977 [135]. The first conjugate vaccine was introduced in 1992. In total, four different conjugate vaccines have been licensed, each with different immunologic properties [136]. In 2012, it was concluded that the invasive disease caused by Hib had been virtually eliminated since the introduction of the vaccine [136, 137]. However, Hib vaccines do not protect against other serotypes. There are currently no approved vaccines against the remaining capsulated *H. influenzae* nor against NTHi, and so research is thus needed [138–140]. For instance, recent studies on the prevention of chronic obstructive pulmonary disease (COPD) focused on the immunogenicity of various vaccine formulations consisting mainly of NTHi and *Moraxella catarrhalis* surface proteins [141].

Two relevant candidate vaccine antigens are the surface proteins Hia and Hsf. Both TAAs contain several repetitive domains, are homologous in their N- and C-termini, and show an overall sequence identity of 81% and 72%, respectively [132].

#### *Haemophilus influenzae* surface fibril

Hsf has a monomeric molecular weight of 243 kDa [132], represents a major virulence factor of *H. influenzae*, and is presented in all encapsulated serotypes and a subset of NTHi [132]. The binding of vitronectin by Hsf inhibits the formation of the membrane attack complex and thus facilitates the invasion of lung epithelial cells [131]. Furthermore, Hsf mediates adherence to host epithelial surface integrins via bridge formation with vitronectin. Hsf is not frequently mentioned as potential vaccine antigen, but Hallström et al. demonstrated reduced survival of a Hsf-deficient mutant when incubated with human serum [129, 142].

#### *Haemophilus influenzae* adhesin

In contrary with Hsf, Hia is only present in 25% of NTHi clinical isolates and has a monomeric molecular weight of 114 kDa [130, 131, 133]. Hia is a major adhesin in NTHi strains and performs a crucial role in the infection and colonisation of the upper respiratory tract [143]. In addition, Hia is highly immunogenic in humans and a strong antibody induction was observed during naturally acquired infections [144, 145]. However, to qualify as a vaccine antigen, a broad strain coverage is required. A vaccine that comprises Hia, combined with both surface adhesins HMW1 and HMW2, would be active against 95% of all NTHi [130, 133, 144, 146]. HMW1 and HMW2 are both immunogenic surface adhesins expressed by approximately 75% of NTHi strains [130, 146]. Winter and Barenkamp demonstrated in 2017 the protective ability of OMVs, overexpressing HMW1 and HMW2 or Hia, as vaccine antigens in a rodent otitis media model [62].

#### *Haemophilus ducreyi* serum resistance A

The TAA of *Haemophilus ducreyi* called the ‘*ducreyi* serum resistance A’ (DsrA) is a proven virulence factor and thus a potential target as vaccine antigen [147]. *H. ducreyi* is a pathogen that causes the genital ulcer disease chancroid, for which no vaccines are available [148]. Fusco et al. demonstrated the immunogenic and protective properties of a recombinant form of the N-terminal passenger domain of DsrA (rNT–DsrA_I_), administered bi-weekly in Freund’s adjuvant against infection with experimental *H. ducreyi* in swine [66]. It was subsequently found that the humoral immune response in mice upon intramuscularl administration of rNT–DsrA_I_ with alum is highly persistent and of superior quality and quantity compared to subcutaneous administration [149]. Furthermore, a Th2-type immune response was observed using Freund’s adjuvant, alum, or imiquimod as adjuvant [149]. Nonetheless, *H. ducreyi* is divided into two clonal populations with varieties in the passenger domain of DsrA, meaning that antibodies recognising class I DsrA do not recognise class II DsrA [147, 149, 150].

### *Acinetobacter baumannii* TAA

Ata is a TAA present on the bacterial surface of the Gram-negative *A. baumannii,* one of the major causative agents of hospital-acquired infections worldwide [151, 152]. Characteristically, *A. baumannii* strains possess the ability to acquire resistance genes rapidly against all commonly used antimicrobial compounds. The dissemination of carbapenem-resistant and in general multidrug-resistant *Acinetobacter* spp. strains is one of the most urgent health risks of our time and threatens to undo a century of medical progress [153]. Consequently, *A. baumannii* is the number one pathogen on the ‘WHO priority pathogens list for R&D of new antibiotics’ [154]. Effective antibiotic treatment is thus complicated and alternative therapy strategies are urgently needed [63, 151, 152, 155].

Vaccination can become a valuable alternative for shortcoming antibiotic treatments against multi-resistant pathogenic strains. Currently, no vaccines against *A. baumannii* are licensed. However, promising vaccine candidates with immunogenic and protective properties have been described, including outer membrane complexes, OmpA and Ata itself [155–157].

Ata was first described in 2012 while searching for novel virulence factors of *A. baumannii* [158]. The *ata* gene was detected in 44 out of 75 collected *A. baumannii* isolates of which 43 showed additional Ata expression on its outer membrane [158]. More recently via phylogenetic profiling, 78% of monophyletic *A. nosocomialis*, *A. seifertii,* and *A. baumannii* showed presence of the *ata* gene [159]. Ata mediates binding to ECM proteins, under static and dynamic flow conditions [160], plays a crucial role in biofilm formation, mediates virulence in vitro and in vivo, and is hence an important virulence factor [63, 158, 159]

Bentancor et al. demonstrated in a pneumonia infection model in immunocompetent and immunocompromised mice the promising bactericidal, opsonophagocytic, and protective features of Ata-induced antibodies against *inter alia* two heterologous unrelated multidrug resistant *A. baumannii* strains [63]. Nevertheless, to increase the efficacy and strain coverage, the combination of Ata proteins from various isolates was suggested. In addition, the use and effectiveness of reverse vaccinology in the search for potential vaccine antigens against *A. baumannii* were recently re-emphasized [55, 156].

### *Moraxella catarrhalis* TAAs

*Moraxella catarrhalis* expresses two different TAAs on its outer membrane, the ubiquitous surface protein A1 (UspA1) and the ubiquitous surface protein A2 (UspA2) [18, 161].

*Moraxella catarrhalis* is a Gram-negative and a human-specific bacterium of the respiratory tract [162, 163]. It was previously classified as *Micrococcus catarrhalis*, *Neisseria catarrhalis,* and *Branhamella catarrhalis* [164]. *M. catarrhalis* is a commensal coloniser of the nasopharynx and represents a causative agent of otitis media in (young) children. The role of *M. catarrhalis* as causative agent of COPD has long been underestimated, however, is a frequent pathogen in the acute exacerbation phase of the disease [141, 165]. Other related illnesses are meningitis, sinusitis and pneumonia [18, 162]. Diseases caused by *M. catarrhalis* are a serious burden for health systems worldwide [166, 167]. Moreover, *M. catarrhalis* produces ß-lactamases and is thus resistant against various important antibiotics [18]. Alternative therapies, such as *M. catarrhalis* vaccines, are, therefore, highly desirable [168].

Currently, no licensed vaccines are available to prevent *M. catarrhalis*-associated diseases, but several candidate vaccines are being developed [165, 168, 169]. Potential *M. catarrhalis* vaccine antigens are adhesive proteins (e.g., OMP CD, *Moraxella* IgD-binding protein, UspA1 and UspA2), proteins involved in nutrient acquisition (e.g., oligopeptide permease protein A, transferrin-binding proteins, and OMP E), lipooligosaccharides, or other *Moraxella* surface proteins [18, 170, 171]. Numerous OMPs including UspA1 and UspA2 are main virulence factors of *M. catarrhalis* and play an important role in the first adherence to the epithelial host cells, during the infection process, and the subsequent disease development [163].

UspA1 and UspA2 are TAAs with a predicted molecular weight of ca. 83.5 and ca. 59.5 kDa, respectively [172]. They are immunogenic [161, 173] and play an important role in serum resistance [174]. In addition, UspA1 and UspA2 are identified as one of the main targets of antibodies to surface epitopes in patients with COPD [175, 176]. Earlier, UspA1 and UspA2 were considered as promising vaccine candidates [18, 171, 173, 177]. However, a high degree of sequencing diversity in the *uspA1* and *uspA2* genes was demonstrated [163, 178] resulting in strain-specific differences and variable phenotypes [179]. In addition, to evade acquired immunity from the host while maintaining serum resistance and adhesive features, regions of *uspA* genes can swap between other *uspA* genes from the same strains [180]. Consequently, both TAAs lately lost major interest as potential vaccine antigens [18]. A possible solution might be to target conserved motifs of known function that are present in both UspA proteins [e.g., domains responsible for binding with ECM proteins or proteins from the carcinoembryonic antigen related cell adhesion molecule (CEACAM) subfamily] [180].

### *Escherichia coli* TAAs

Four different TAAs have been characterised from pathogenic *Escherichia coli*, in particular the *E. coli* immunoglobulin binding (Eib) proteins [181], the Shiga toxin-producing *E. coli* auto-agglutinating adhesin (Saa) [182], the uropathogenic *E. coli* adhesin G (UpaG) [183], and, most recently, the enterohemorrhagic *E. coli* adhesin G (EhaG) [184].

Currently, no broadly protective vaccines against pathogenic *E. coli* are available [185, 186], but some vaccines have reached clinical trial status [187–189]. Most research concerning vaccine development against pathogenic *E. coli* is done for the enterotoxigenic *E. coli* (ETEC) expressing enterotoxins and colonisation factors (i.e., usually fimbriae or fibrillae) upon infection [190], as this bacterium is an important cause of bacterial diarrhoea (travellers’ diarrhoea) in developing and middle-income countries [187]. ETEC vaccine development is currently one of the WHO priorities [191, 192]. Two vaccines against ETEC are in phase II clinical trials. To broaden the vaccine coverage, novel immunogenic, conserved and virulent antigens must be reviewed, e.g., non-fimbrial surface adhesins [193]. Promising research to identify potential protective antigens is ongoing [185, 194–196].

#### *Escherichia coli* immunoglobulin binding proteins

Eib proteins are mostly found in intimin-negative, shiga toxin-producing enterohaemorrhagic *E. coli* (EHEC) strains [197, 198]. Shiga toxin-producing *E. coli* (STEC) causes severe diseases in humans such as haemorrhagic colitis or haemolytic–uremic syndrome (HUS) [199]. *Eib* genes occur in various pathogenic and multidrug-resistant *E. coli* strains, for example enteroaggregative *E. coli* (EAEC), extraintestinal pathogenic *E. coli* (ExPEC), and verotoxigenic *E. coli* (VTEC) [199–201]. No licensed vaccines against STEC-associated diseases are available [196].

Currently, six homologous Eib proteins are described (EibA, EibC, EibD, EibE, EibF, and EibG) [181, 197, 198]. They are all TAAs and mutually share a high similarity in their passenger domain and C-terminus. Eib proteins are major virulence factors, as they (i) mediate serum resistance; (ii) play a major role in adherence to epithelial cells; and (iii) are receptors for IgAs and IgGs, binding non-immunologically to the Fc portion of immunoglobulins (Ig) [197, 198, 202]. To the best of our knowledge, no research has been carried out on their potential as vaccine components.

#### Shiga toxin-producing *E. coli* auto-agglutinating adhesin

In 2001, the gene for Saa was isolated from a large, virulence-related plasmid in a STEC strain negative for the locus for enterocyte effacement. Saa mediates autoaggregation and adherence to human epithelial type 2 cells, shows variation in size for different STEC strains, and has just ca. 25% identity with the Eib proteins. Furthermore, Saa was not proven to contribute to serum resistance. Nevertheless, in vitro adherence of *saa*-positive STEC strains was inhibited upon application of a polyclonal antiserum that was raised against purified Saa, emphasizing its potential as a vaccine antigen [182].

#### Uropathogenic *E. coli* adhesin G

*Escherichia coli* UpaG, was identified by Durant et al. via reverse vaccinology [195]. UpaG, characterised in the uropathogenic *E. coli* (UPEC) strain CFT073, mediates binding to ECM proteins and bladder epithelial cells, and promotes bacterial cell aggregation and biofilm formation [183]. The *upaG* gene in UPEC shows extensive sequence variation with the *upaG* gene in ExPEC strains [184].

Furthermore, UpaG was proven to induce protective antibodies in a mouse model against lethal sepsis due to virulent extraintestinal isolates of *E. coli* [195]. UpaG shows a wide strain distribution and is present in both commensal and pathogenic strains (e.g., in ExPEC strains) [203], suggesting that it is important in efficient colonisation of the urinary tract [183].

#### Enterohaemorrhagic *E. coli* adhesin G

The most recently identified TAA is EhaG which occurs in EHEC strains. EhaG is a positional orthologue of UpaG [184, 204], but has significant sequence differences in the passenger domain and has some divergent functional characteristics. EhaG also mediates bacterial binding to ECM proteins, autoaggregation, and biofilm formation. Other than UpaG, EhaG promotes adherence to intestinal epithelial cells. In addition, EhaG is highly conserved in diarrheagenic *E. coli* strains [184]. Some of these features indicate that EhaG is suitability as a potential vaccine candidate, but more research on it is certainly necessary.

### *Salmonella enterica* adhesin A

*Salmonella* adhesin A (SadA) is a TAA expressed in vivo on the bacterial surface of the pathogenic *Salmonella enterica* (serovar Typhimurium) during infections [64, 205].

*Salmonella enterica* causes significant morbidity and mortality worldwide in humans and cattle [206]. Moreover, *Salmonella* is the most frequent bacterial cause of foodborne disease in the US and is responsible for the majority of foodborne outbreaks in the European Union [207]. Infection with *S. enterica* can result in enteric salmonellosis and sometimes manifests as septicaemia. When not self-limiting, *Salmonella*-infected patients are treated via antimicrobial therapy. Consequently, multidrug-resistant *S. enterica* are on the rise [206, 208].

Currently, three types of licensed *Salmonella* vaccines exist: (i) a whole-cell live-attenuated vaccine (Vivotif^®^); (ii) a polysaccharide unconjugated vaccine; and (iii) a polysaccharide-conjugated vaccine (the latter commercialised under several names), all against one *S. enterica* serovar Typhi [209]. Furthermore, vaccination therapy against *Salmonella* spp. does exist for livestock breeding; for instance, an attenuated *S. enterica* serovar Typhimurium was designed providing higher cross protection against *Salmonella* serovars in swine [210]. Despite various studies and existing vaccines, there is still a need for safer and well-defined *Salmonella* vaccines.

The TAA SadA has an approximate trimeric size of 426 kDa and promotes biofilm formation and autoaggregation, but does not mediate serum resistance and does not bind ECM proteins. In addition, no distinction in virulence was observed between wild-type *S. enterica* and SadA-deficient *S. enterica*. SadA, nonetheless, plays an important role in adherence to and invasion of intestinal epithelial cells. Large surface structures such as LPS or fimbria inhibit the function of SadA, suggesting a specific role during certain conditions in colonisation and infection of epithelial cells. Moreover, SadA is highly conserved within *S. enterica* strains and is considered as a positional orthologue of UpaG and EhaG in *E. coli,* but with some different functions [64, 204]. An immunological IgG response was observed upon immunisation of mice with purified SadA (and Alum as adjuvant). However, IgG antibodies to SadA give only a limited protection compared to the PBS control, and therefore, the development of an effective vaccine against *S. enterica* might involve multiple antigens in parallel [64].

### *Bartonella* TAAs

*Bartonella quintana*, *B. bacilliformis,* and *B. henselae* are clinically the three most important *Bartonella* species each expressing one or more TAAs [211, 212]. *B. quintana* expresses four variably expressed outer membrane proteins (VompA–D) [213], *B. bacilliformis* the *B. bacilliformis* adhesins A, B, and C (Brps, also designated as BbadA–C) and *B. henselae* the *B. henselae* adhesin A (BadA). Antimicrobial treatment of *Bartonella* spp.-associated diseases depends solely on the clinical situation and immunological status of the patient and less on the infective species. Consequently, no general treatment recommendation does exist for all *Bartonella* spp.-associated diseases [212, 214].

#### Variably expressed outer membrane proteins

*Bartonella quintana* is transmitted via the human body louse and is the causative agent of trench fever. Infections with *B. quintana* can lead to endocarditis, bacillary angiomatosis and peliosis hepatis in immunocompromised patients [211, 215–217]. Until now, no vaccines exist or are being developed against *B. quintana* infections [212].

*Bartonella quintana* expresses four TAAs called VompA–D, which are encoded by four genes (*vompA, vompB, vompC,* and *vompD*) and are tandemly arranged in a 12.8 kb gene locus [213]. The domain structure of the four ca. 100 kDa VompA–D is highly conserved, except for the major variable region in the N-terminal half of the stalk [213]. This region might be responsible for the variable phenotypes amongst the VompA–D which causes diversity in adhesion specificity, e.g., expression of VompA mediates autoaggregation of *B. quintana* [218]. Vomps are involved in bacterial cell adhesion to endothelial HUVEC cells [219], but do not seem important for bacterial adherence to epithelial HeLa-229 and phagocytic THP-1 cells [215]. Vomps are, therefore, important virulence factors and are crucial for the course of infection [213, 218].

The immunogenicity of Vomps and their suitability as a vaccine antigen have been described. While analysing protective and diagnostically relevant *B. quintana* antigens, 24 immunoreactive membrane proteins were identified of which, among others, VompA and VompB were most frequently recognised by sera from *B. quintana*-infected patients [220]. Further research to classify both TAAs as vaccine antigens is, however, necessary.

#### *Bartonella bacilliformis* adhesins A–C

*Bartonella bacilliformis* is the causative agent of Carrion’s disease, a biphasic illness restricted to the South American Andes [221]. The pathogen can infect human erythrocytes causing a serious acute hemolytic anaemia called ‘Oroya fever’ with high mortality rates in untreated patients. In a second chronic phase, *B. bacilliformis* infects endothelial cells and stimulates cell proliferation which results in the formation of blood-filled nodular haemangioma-like lesions in the skin known as ‘verruga peruana’ [221].

Currently, no vaccine is available for *B. bacilliformis*. However, vaccines against *B. bacilliformis* infections should be effective, as indigenous people in *B. bacilliformis* endemic regions seem less susceptible to infections and hemolytic diseases compared to non-indigenous people [222]. In addition, antiflagellin antiserum significantly reduced in vitro human erythrocyte invasion by the pathogen as compared to the controls [223].

One of the few attempts to prepare a vaccine against the Carrion’s disease was performed in 1943 by Howe and Hertig. The vaccine contained a formalin suspension of four *B. bacilliformis* strains. Twenty-two Peruvian guards working in a region notorious for frequent incidents of Carrion’s disease were subcutaneously vaccinated. The vaccine did not prevent infection, but alleviated the severity of the Carrion’s disease [221, 224]. Nonetheless, as the highly deadly Carrion’s disease affects mostly indigenous people with only limited medical care, the most promising and effective strategy to fight this disease is the development of a vaccine evoking both humoral and cellular immune responses [221, 225, 226].

In *B. bacilliformis*, three genes encoding for putative TAAs were identified [211]. The *B. bacilliformis* adhesins A–C (BbadA–C), originally called *Bartonella* repeat proteins (Brps), share common domain features with other TAAs of the genus *Bartonella*. The 130 kDa monomeric BbadA shows a highly similar head structure compared to BadA of *B. henselae*. In contrast, BbadB and the much shorter BbadC have more in common with the VompA–C of *B. quintana*. The role of *B. bacilliformis* adhesins during the infection process remains unclear.

Among other candidates [226, 227], the TAAs BbadA–C have been described as potential antigen candidates in vaccines [226]. More research towards antigenic candidates is, however, necessary and ongoing.

#### *Bartonella henselae* adhesin A

*Bartonella henselae* is the etiologic agent of cat scratch disease (CSD) and vasculoproliferative disorders. CSD is a self-limiting disease, but can be life-threatening for immunocompromised patients [29, 211]. Cats and dogs are the main reservoir of *B. henselae*, and the role of ticks in the transmission of *B. henselae* to humans remains unclear [29, 228, 229].

Unlike research towards clinical serodiagnostic tools including immunogenic proteins of *B. henselae* [230–233], and towards the development of feline vaccines [234–237], there has been no research towards the development of human vaccines preventing *B. henselae* infections. One possible obstacle in this research is the variable gene pool of *B. henselae* strains promoting antigenic variation and defining the specific immune response [238].

An important pathogenicity factor of *B. henselae* is the TAA BadA. BadA is a large (ca. 240 nm and ca. 328 kDa monomeric) outer membrane protein primarily responsible for the first interaction of the pathogen with endothelial host cells and ECM proteins (e.g., collagen, fibronectin, and laminin) (Fig. 2). Expression of BadA correlates with the secretion of angiogenic cytokines and activation of hypoxia-inducible factor (HIF)-1, the key transcription factor involved in angiogenesis [24, 219, 239, 240]. Despite length variations in the neck-stalk region, BadA seems to be highly conserved within *B. henselae* strains [241].

BadA is an immunodominant and immunogenic protein and regularly found in sera of patients (75%) infected with *B. henselae* [231, 240]. A mixture of immunodominant proteins including BadA seems the most plausible approach to develop an effective vaccine [232, 238].

### Other TAAs

The *Brucella suis* trimeric autotransporter F (BtaF) and E (BtaE) are described as a promising immunogenic target for vaccination against mucosal *B. suis* infections [242]. Other research concerning the vaccine development targeting melioidosis and glanders caused by *Burkholderia pseudomallei* and *Burkholderia mallei*, respectively, is ongoing and describes various expressed TAAs with immunogenic properties such as BPSL2063 [243] and BimA [244, 245].

Finally, vaccine development against animal pathogenic bacteria expressing TAAs is of high veterinary importance. For example, AhsA (designated according to gene locus *ahsA*) of *Mannheima haemolytica* A1, the principal cause of bovine pneumonic pasteurellosis, promotes colonisation and subsequent infection via its ability to bind collagen and, more importantly, is suggested to be immunogenic in calves [246]. Furthermore, the TAA HMTp210 is the major hemagglutinin antigen of *Avibacterium paragallinarum*, which can cause infectious coryza in poultry. A variable region within HMTp210 is proposed as a candidate for recombinant vaccine production [247–249]. Lastly, *Actinobacillus pleuropneumoniae* adhesin 1 (Apa1) and 2 (Apa2) are two TAAs expressed on the bacterial surface of *Actinobacillus pleuropneumoniae*, the causative agent of porcine pleuropneumonia. The main functional head domain, Apa2H1, activates dendritic cells and provides effective protection in mice against lethal infections with *A. pleuropneumoniae* by both reducing bacterial colonisation and dissemination [250].

## Conclusion

Remarkably, it is still only NadA of all TAAs that is used as a main vaccine antigen in the respective multicomponent vaccine 4CMenB. Nonetheless, TAAs largely fulfil the requirements to be considered as potential vaccine antigens. Immunogenicity was demonstrated for many of the TAAs (Table 1). Moreover, seven out of nine already assessed TAAs (YadA, NadA, DsrA, Ata, UspA1-2, UpaG, and SadA) induced a protective host response upon infection with the respective pathogen.

The reason for this ‘scarcity’ of TAAs as vaccine (sub)units may be that research on TAAs itself is still fairly new, especially research towards their applicability as potential vaccines. The current trend to use OMVs in vaccines (as in 4CMenB) and to apply reverse vaccinology to identify new vaccine antigens might give a boost for the usage of TAAs as vaccine antigens.

TAAs showing the highest potential as vaccine targets are Hia of *H. influenzae*, DsrA of *H. ducreyi*, Ata of *A. baumannii*, UpaG of uropathogenic *E. coli* and EhaG of enterohaemorrhagic *E. coli*. TAAs that are no longer of major interest as vaccine targets are NhhA of *N. meningitidis* and UspA1 and UspA2 of *M. catarrhalis* due to their irregular expression patterns and high degree of diversity, respectively. Other TAAs, including YadA of *Y. enterocolitica*, Saa of Shiga toxin-producing *E. coli*, BbadA–C of *B. bacilliformis,* and VompA and VompB of *B. quintana* show promising results as potential future vaccine candidates. In conclusion, more extensive research bringing more insights in the functionality and effectiveness of TAAs as vaccines is necessary.
